# Metabolic Signatures of Four *Polygonatum* Rhizoma Species Mapped Using Untargeted Metabolomics

**DOI:** 10.3390/metabo15110682

**Published:** 2025-10-22

**Authors:** Ning Jia, Jinlan Jiang, Wei Ye, Jiqin Liu

**Affiliations:** 1 College of Agricultural and Forestry Science and Technology, Hebei North University, Zhangjiakou 075131, China; 2 Fujian Key Laboratory of Crop Genetic Improvement and Innovative Utilization for Mountain Area, Sanming Academy of Agricultural Sciences, Sanming 365050, China; fjsxjjl@163.com (J.J.); yewei922@163.com (W.Y.); 3 Institute of Plant Quarantine, Science and Technology Research Center of China Customs, Beijing 100026, China

**Keywords:** untargeted metabolomics, *Polygonati* rhizoma, LC-MS/MS, triterpenoid, polysaccharide, traditional Chinese medicine

## Abstract

Background/Objectives: *Polygonati* rhizoma (PR) is a common traditional Chinese medicine that has been used for 2000 years in China, serving as both food and medicine. It is known for various health benefits, including antidiabetic effects, regulation of gut microbiota, and enhancement of immunity. The most popular PR varieties are *Polygonatum kingianum* Coll. et Hemsl. (PK), *Polygonatum sibiricum* Red. (PS), *Polygonatum cyrtonema* Hua (PC), and *Polygonatum odoratum* (Mill.) Druce (PO). We aimed to determine the differences among these four PR species. Methods: Using an untargeted mass spectrometer we conducted a metabolomic analysis. Results: We detected 2360, 2336, 2381, and 2355 unique polysaccharide, steroid, alkaloid, nucleoside, and peptide metabolites, among which 10, 36, 5, and 26 were specific to PK, PS, PC, and PO, respectively. Differentially expressed polysaccharide, steroid, and alkaloid metabolites were identified in the four species. A total of 61, 56, 61, and 57 carbohydrates were identified in the PK, PS, PC, and PO, respectively; 33, 32, 29, and 30 steroids were identified in the PK, PS, PC, and PO, respectively; and 10, 12, 12, and 11 alkaloids were identified in PK, PS, PC, and PO, respectively. Conclusions: Our findings provide novel insights into the overall metabolome of the four PR species, improve understanding of their functions and effectiveness, and provide a theoretical basis for qualitative evaluation and comprehensive PR applications.

## 1. Introduction

*Polygonati* rhizoma (PR) is a medicinally valuable traditional Chinese medicine, published more than 2000 years ago in the MingYi BieLu [1]. PR improves immune function and is a common raw material and food source in medicinal Chinese cuisine [2,3]. Therefore, PR has been considered a “medicine food homology” by the National Health Commission of the People’s Republic of China [4]. More than 60 species of the *Polygonatum* genus have been documented, and they are widely distributed in subtropical, temperate, and cold–temperate zones in the Northern Hemisphere. Approximately 39 of these species naturally occur in China. In the *Chinese Pharmacopoeia*, dry rhizomes of the *Liliaceae Polygonatum* genera primarily include *Polygonatum kingianum* Coll. et Hemsl. (PK), *Polygonatum sibiricum* red. (PS), *Polygonatum cyrtonema* Hua (PC), and *Polygonatum odoratum* (Mill.) (PO) [5]. PR contains various functional carbohydrates, steroids, alkaloids, nucleosides, and peptides [6]. Studies of the clinical and molecular mechanisms of Chinese medicines have indicated that PR has good antihyperglycemic [7,8], anti-inflammatory [9], immune regulatory, blood lipid-lowering, and antitumor effects, with potential pharmaceutical and medical value [10]. Recently, with the increased awareness of health improvement, PR has become a significant research hotspot [11].

Several studies on PR have focused on chemical components, such as steroid saponins, carbohydrates, and flavonoids. Current literature has mostly focused on the characteristic and primary active steroid saponins, with only one study reporting monomeric saponins [12]. To date, 67 steroid saponins belonging to 11 classes have been isolated from PK, PC, and PS. Triterpene saponins are among the most significant PR chemical components, of which 12 types have been isolated from PK and PS. Another characteristic of PR is its high isoflavone content. To date, three isoflavones [13], two dihydroflavonoid neo glycyrrhizins [14], two chalcone isoliquiritins [14], two neoisoglycyrrhizins and three 4′,7-dihydroxy-3′-methoxyisoflavones [15] have been isolated from PR. However, the alkaloid content in PR is relatively low. Five alkaloids including adenosine and N-trans-p-coumaroyloctopamine have been identified [16]. Among these compounds, new indoleazine alkaloids were detected, which are the basic nuclei of a hexa-penta-heterocyclic system containing nitrogen.

Polysaccharides have emerged as the primary PR metabolites and are relatively abundant in *Polygonatum,* with their content ranging from 4.47% to 21.34% [17]. Liu et al. isolated the neutral polysaccharides, PSW-1a and PSW-1b-2, from PR [18]. The *Polygonatum* polysaccharide PSP I, whose main chain comprises fructose linked by β-(1→2) bonds, was also isolated from PR [19]. The three polysaccharide components isolated from *Polygonatum yunnanensis*, are PKP I–III, of which PKP I contains approximately 50 glucose units [20]. Five *Polygonatum* polysaccharides have been isolated, and their physical properties and chemical structures have been characterized using FT-IR and NMR [21]. In addition, 21 common differential metabolites, including lipid and lipid-like molecules, benzenoids, lignans, organic acid and derivatives, phenylpropanoids and polyketides, and neolignans or related compounds, were identified in one wild and three cultivated PR species [22]. The composition of protein, polysaccharide, lipid, isoflavone, flavonoid, and saponin metabolites in PR changes depending on the processing [23]. The content of saccharide and organic acid metabolites increases after nine cycles of steaming and drying in the sun, whereas that of amino acids and their derivatives, alkaloids, and terpenoids decreases [24].

This study aimed to determine the differences in metabolites among PK, PS, PC, and PO. We conducted an untargeted metabolomic analysis using a liquid–mass spectrometry system comprising a UHPLC ultra-performance liquid phase tandem QE high-resolution mass spectrometer. We initially determined the differential metabolites between the four PRs using statistical analysis and further explored the relationships among the metabolites through correlation and clustering analyses. We then investigated the differential metabolites among the four PR through pathway enrichment analysis using the Kyoto Encyclopedia of Genes and Genome (KEGG) database. Our findings provide insights into the chemical composition and characteristics of four PRs and offer a theoretical foundation for their application to treating specific diseases.

## 2. Materials and Methods

### 2.1. Sample Collection

**Figure 1:**
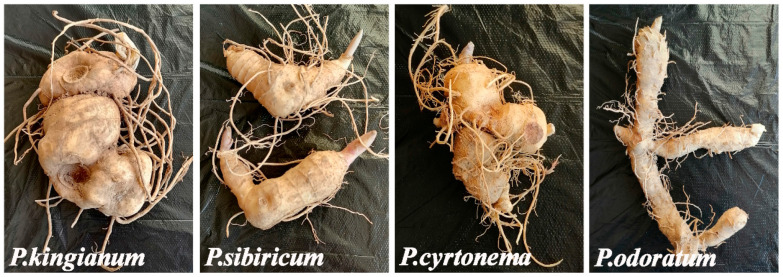
Two-year-old rhizomes from four *Polygonatum* species.

*Polygonatum sibiricum* Red. (PS) was collected from Gaoying Village, Hanzhong City, Shanxi Province (longitude 107°3′19″ E; latitude 33°6′38″ N). *Polygonatum cyrtonema* Hua (PC) was collected from Baoshi, Xinqiao Township, Taining County, Sanming City, Fujian Province (longitude 117°10′35″ E; latitude 27°4′78″ N). *Polygonatum kingianum* Coll. et Hemsl. (PK) was collected from Maguan County, Wenshan City, Yunnan Province (longitude 104°9′30″ E; latitude 23°3′59″ N). *Polygonatum odoratum* (Mill.) Druce (PO) was collected from Dachongzi, Anhua County, Yiyang City, Hunan Province (longitude 111°19′40″ E; latitude 28°34′23″ N). Samples were packaged in dry ice and transported to the laboratory (Figure 1).

### 2.2. Sample Preparation and Liquid Chromatography-Tandem Mass Spectrometry (LC-MS/MS)

Liquid chromatography/mass spectrometry (LC/MS) analysis of the extract was conducted by Luming Bio Co. (Shanghai, China). PR powder samples (80 mg) were added to 20 μL of internal standard (L-2-chlorophenylalanine, 0.3 mg/mL; methanol) and 1 mL of methanol:water (7:3 *v*/*v*). Sample tubes were placed at −20 °C for 2 min. The samples were extracted for 30 min in an ice water bath, and stored at −20 °C overnight. The samples were centrifuged at 13,000× *g* at 4 °C for 10 min. Supernatants (150 μL) were collected, passed through a 0.22-μm organic phase filter, and stored in LC sample bottles at −80 °C for LC-MS.

All samples were analyzed six times using a Dionex UltiMate U3000 UHPLC ultra-performance liquid-phase tandem QE high-resolution mass spectrometer. The samples were separated using an ACQUITY UPLC HSS T3 chromatography column (100 × 2.1 mm, 1.8 μm). The mobile phases A and B comprised ultrapure water and acetonitrile containing 0.1% formic acid, respectively. The column temperature was maintained at 45 °C. The flow rate was 0.35 mL/min, and the injection volume was 2 μL. The elution profile was 0–2 min, 5% B; 4 min, 30% B; 8 min, 50% B; 10 min, 80% B; 14 min, 100% B; 15 min, 100% B; 15.1 min, 5% B; 18 min, 5% B, and the ion source was electrospray ionization (ESI). The quality of spectrum signals in the samples was assessed using positive and negative ion modes. The mass range was 100–1000 *m*/*z*. The resolution was set at 70,000 for the full MS scans and 17,500 for HCD MS/MS scans. Collision energy was set at 10, 20, and 40 eV. The mass spectrometer operated as follows: spray voltage, 3800 V (+) and 3200 V (−); sheath gas flow rate, 35 arbitrary units; auxiliary gas flow rate, 8 arbitrary units; capillary temperature, 320 °C; Probe heater temperature, 350 °C; S-lens RF level, 50.

### 2.3. Processing and Analysis of UPLC-MS/MS Test Data

The original data were preprocessed before pattern recognition using the Progenesis^®^ QI v2.3 metabolomic analysis software (Waters Corporation, Milford, MA, USA and Nonlinear Dynamics, Newcastle upon Tyne, UK), followed by baseline filtering, nonlinear dynamics, integration, retention time correction, peak alignment, and normalization. The primary parameters were precursor tolerance (5/10 ppm), product tolerance (10/20 ppm), and production thresholds (5%). Metabolites were quantified based on precise mass numbers, secondary fragments, and isotope distribution combined with LipidMaps (v2.3), the METLIN and Human Metabolome databases, and an in-house custom database. Peaks in the group with > 50% of missing values (ionic strength = 0) were removed and zero values were replaced with half of the minimum value. The extracted data were processed and screened according to the qualitative findings of the compounds. Those with scores < 36 out of 60 were removed, and positive and negative ion data were combined into a data matrix.

The matrix was assessed using principal component analysis (PCA) in R (factoextra), and the stability of the entire analytical process and the overall distribution among the samples were determined. Differences in metabolites between samples were identified using partial least squares discriminant analysis (PLS-DA) and orthogonal partial least squares discriminant analysis (OPLS-DA). The 200 Response Permutation test and 7-fold cross-validation were used to evaluate the quality of the model. The overall contribution of each variable to group discrimination was ranked according to the projected importance (VIP) value of the V variable in the OPLS-DA model. Significant differences in metabolites between groups were verified using two-tailed Student’s *t*-tests. Differential metabolites with *p* < 0.05 and VIP > 1.0 were screened for extended KEGG pathway enrichment analysis (http://www.genome.jp/kegg/, accessed on 23 January 2024).

### 2.4. Metabolite Correlation Network Construction

Correlations among metabolites were determined using the cor function in R. Linear correlations between two quantitative variables and between different metabolites were measured using Pearson correlation coefficients. Differences in metabolite expression were visually analyzed based on the VIP value.

## 3. Results

### 3.1. PK, PS, PC, and PO Metabolic Profiles

**Figure 2:**
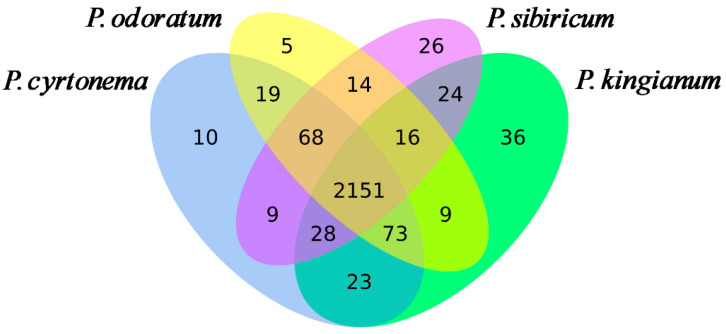
Venn plot of differentially expressed metabolites among *Polygonatum kingianum* (PK), *Polygonatum sibiricum* (PS), *Polygonatum cyrtonema* (PC), and *Polygonatum odoratum* (PO).

We analyzed the metabolic changes in PK, PS, PC, and PO via nontargeted metabolomics using LC-ESI-Q-TOF. We detected and quantified 2360, 2336, 2381, and 2355 unique metabolites in PK, PS, PC, and PO, respectively (Figure 2 and Supplemental Table S1). Although the metabolites of the four PRs significantly overlapped, PK, PS, PC, and PO contained 36, 26, 10, and 5 unique components, respectively (Figure 2, Supplemental Table S1). PC had more unique characteristics than the other three varieties. The specific metabolites of these four species could help distinguish different varieties and provide targets for future studies.

### 3.2. PK, PS, PC, and PO Differential Metabolites Revealed Through PCA and OPLS-DA

**Figure 3:**
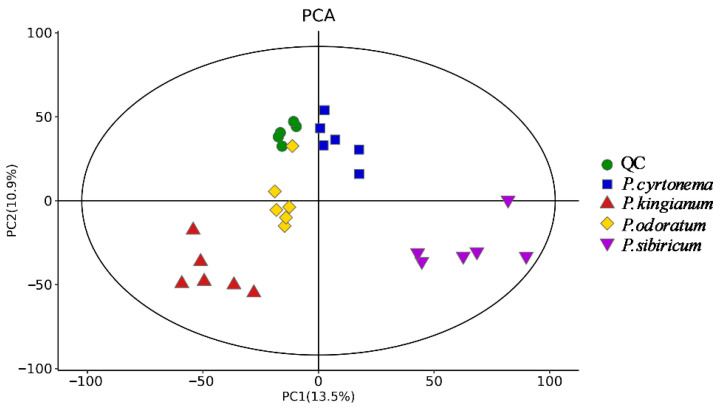
Principal component analysis: PK, PS, PC, and PO scores.

**Figure 4:**
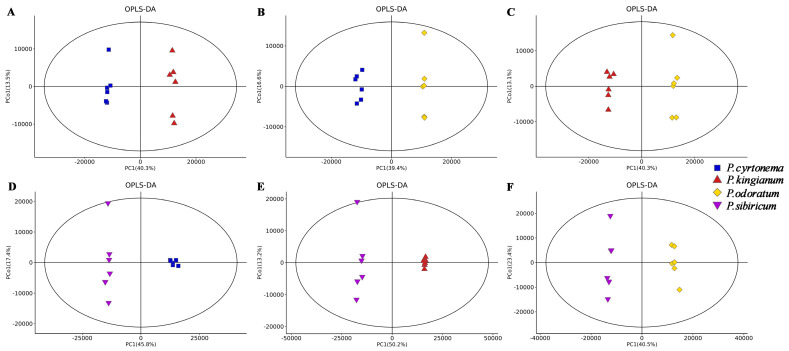
Orthogonal partial least squares discriminant analysis (OPLS-DA): PK, PS, PC, and PO scores. (**A**) OPLS-DA of PC and PK. (**B**) OPLS-DA of PC and PO. (**C**) OPLS-DA of PK and PO. (**D**) OPLS-DA of PS and PC. (**E**) OPLS-DA of PS and PK. (**F**) OPLS-DA of PS and PO.

The PCA results showed that metabolites varied among PK, PS, PC, and PO as they were distributed in different areas (Figure 3). We evaluated the statistical significance of the signals using the OPLS-DA model, and excluded possible confounding variables to analyze differences among PK, PS, PC, and PO. We observed good separation among the four samples, indicating significantly differential metabolites (Figure 4). The OPLS-DA characteristics are presented in Table 1. The values R^2^X > 0.61, R^2^Y > 0.99, and Q2 > 0.85 indicated that this model has good predictive ability. To further understand the metabolites that significantly differed among PK, PS, PC, and PO, those with VIP > 1 and *p* < 0.05 were identified as differentially expressed (Supplemental Table S2).

### 3.3. Characteristic Features of Carbohydrates in PK, PS, PC, and PO

**Figure 5:**
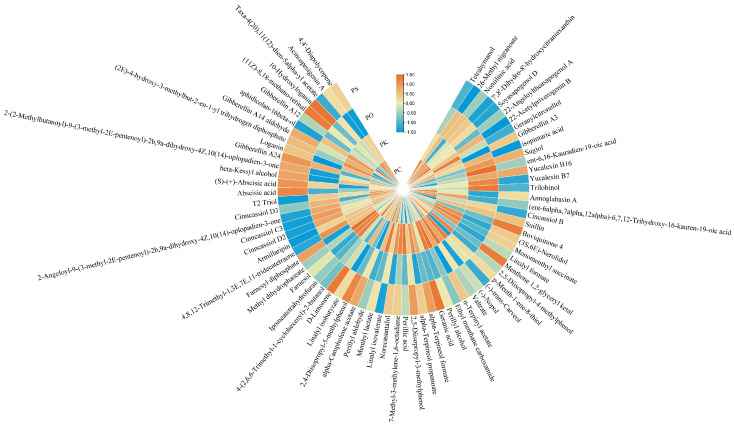
Heatmap of carbohydrates in PK, PS, PC, and PO.

We identified 61, 56, 61, and 57 carbohydrates in the PK, PS, PC, and PO fractions, respectively (Figure 5). Thirteen carbohydrates including beta-lactose, D-ribose, and D-ribulose-5-phosphate, were upregulated, with amylopectin and torachrysone 8-(6-oxalylglucoside) expressed only in PC. Fifteen carbohydrates including galactaric acid, gluconasturtiin, and D-erythrose-4-phosphate, were upregulated in PK, with galabiose expressed only in PK. Twelve carbohydrates, including D-apiose, glucose-6-phosphate, and D-glucono-1,5-lactone 6-phosphate, were upregulated in PO. Twenty-three carbohydrates including (-)-erythro-anethole glycol-1-glucoside, D-glucuronic acid, and lusitanicoside, were upregulated in PS.

### 3.4. Characteristic Features of Steroids in PK, PS, PC, and PO

**Figure 6:**
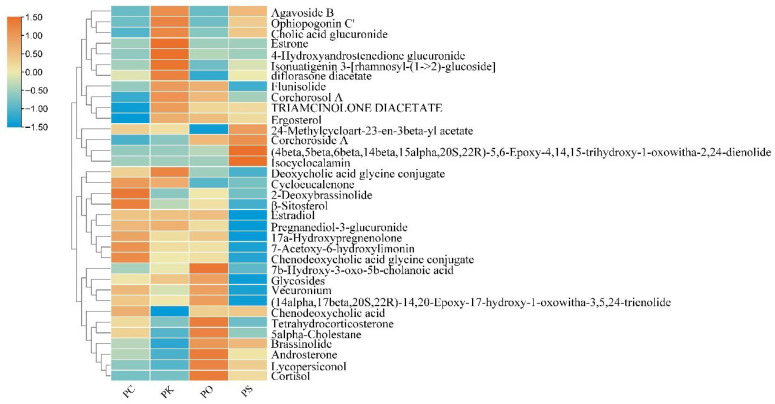
Heatmap of steroids in PK, PS, PC, and PO.

Steroids are significant active constituents of *Polygonatum*. We identified 33, 32, 29, and 30 steroids in the PK, PS, PC, and PO samples, respectively. The expression of steroids among the *Polygonatum* species differed significantly (Figure 6). Cycloeucalenone, 2-deoxybrassinolide, β-ditosterol, 17α-dydroxypregnenolone, 7-acetoxy-6-hydroxylimonin, chenodeoxycholic acid glycine conjugate, and chenodeoxycholic acid were upregulated in PC. The expression of 13 steroids, including agavoside B, ophiopogonin C, cholic acid glucuronide, and estrone, was high in PK, whereas estrone was only expressed in PK. Eleven steroids, including 7β-hydroxy-3-oxo-5b-cholanoic acid, glycosides, vecuronium, and tetrahydrocorticosterone, were upregulated in PO. We also found that 24-methylcycloart-23-en-β-yl acetate, corchoroside A (4β,5β,6β,14β,15α,20S,22R)-5,6-epoxy-4,14,15-trihydroxy-1-oxowitha-2,24-dienolide, and isocyclocalamin) were upregulated in PS, among which the latter was expressed only in PS. By contrast, the expression of other steroids was low in PS.

### 3.5. Characteristic Features of Alkaloids in PK, PS, PC, and PO

**Figure 7:**
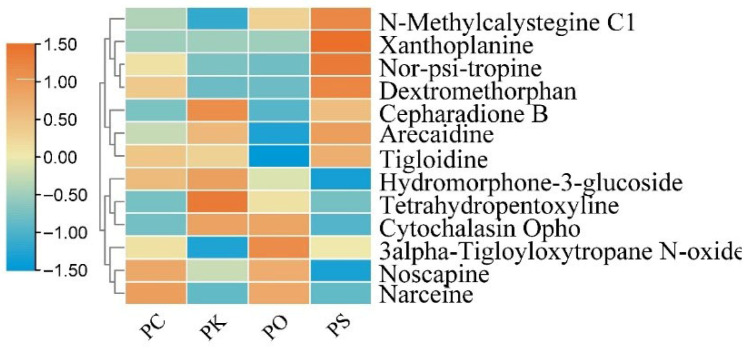
Heatmap of alkaloids in PK, PS, PC, and PO.

Alkaloids are the primary and secondary metabolites in plants. We identified 10, 12, 12, and 11 alkaloids in PK, PS, PC, and PO, respectively, comprising tetrahydro pentoxifylline, arecaidine, nor-psi-tropine, noscapine, tigloidine, hydromorphone-3-glucoside, 3-alfa-tigloyloxytropane N-oxide, N-methylcalystegine C1, cepharadione B, and cytochalasin Opho. Their expression differed among the four *Polygonatum* species (Figure 7). We detected xanthoplanine only in PS, narceine in PC and PO, and dextromethorphan in PS and PC.

### 3.6. KEGG Enrichment of Metabolites That Differed in PK, PS, PC, and PO

**Figure 8:**
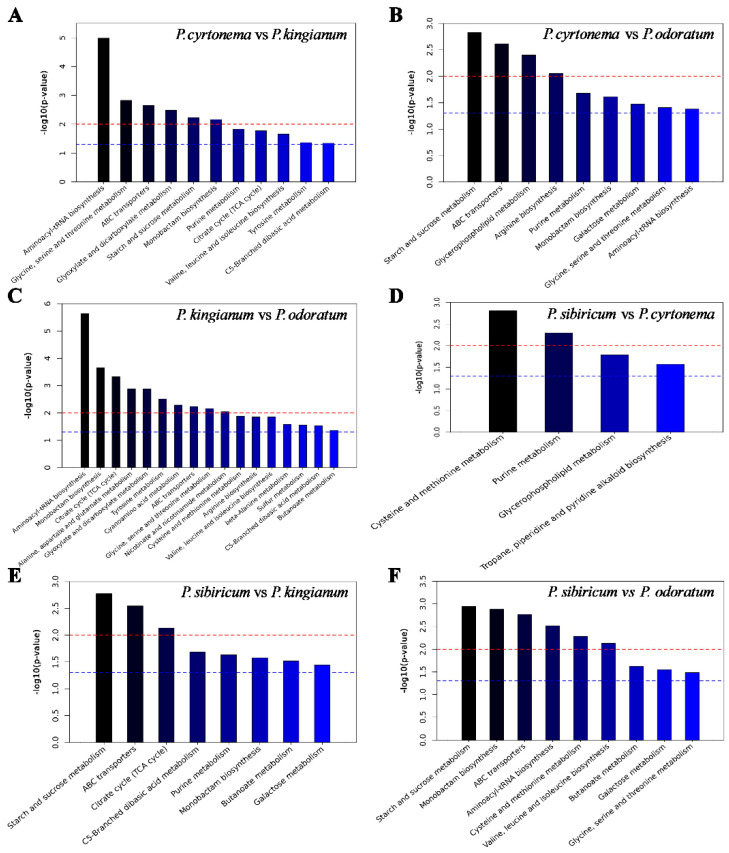
Kyoto Encyclopedia of Genes and Gene (KEGG) pathway enrichment analysis of differentially expressed metabolites among the four Polygonatum rhizoma species. Concentrated *p* value calculated from hypergeometric distribution. *p* = 0.05 was used as a cut-off to reduce FDR. KEGG pathway enrichment comparisons between (**A**) PC and PK, (**B**) PC and PO, (**C**) PK and PO, (**D**) PS and PC, (**E**) PS and PK, and (**F**) PS and PO.

We mapped the PK, PS, PC, and PO metabolites to the KEGG database to determine the metabolic pathways in the four *Polygonatum* species (Figure 8). The differences in metabolic pathways were the lowest between PS and PC, whereas they were the highest between PK and PO. The metabolic pathways of the four species involved lipids, carbohydrates, and amino acids. The metabolism of glycerophospholipids, which are involved in lipid biosynthesis, significantly differed among the four species. The expression of lipid metabolites was the highest in PK and PO, whereas it was the lowest in PS. The metabolic pathways of starch, sucrose, and galactose carbohydrates differed significantly. We identified several amino acid metabolic pathways, including glycine, serine, and threonine metabolism, as well as aminoacyl tRNA, valine, leucine, and isoleucine biosynthetic pathways. The aminoacyl-tRNA biosynthetic pathway is closely associated with protein metabolism.

### 3.7. Species-Specific Diagnostic Markers

**Figure 9:**
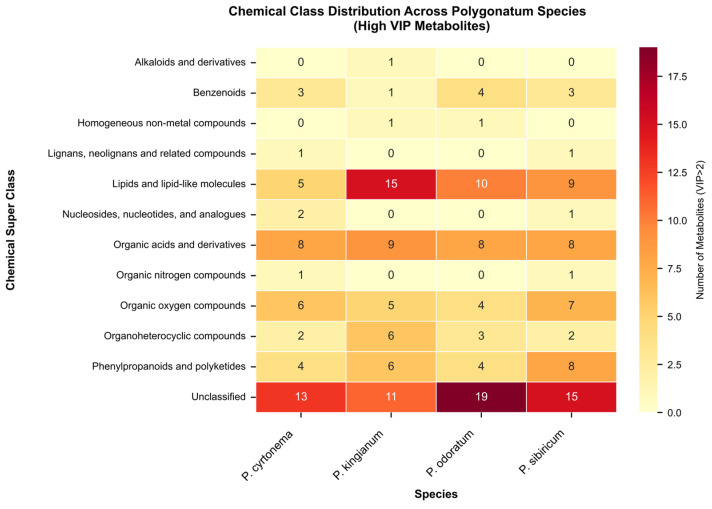
Heatmap of chemical class of top diagnostic metabolites across Polygonatum rhizoma species.

Systematic identification of diagnostic markers across all pairwise comparisons revealed 297 distinctive metabolites (Supplementary Table S3 and Figure 9), of which 168 were species-specific. PK showed 71 exclusive markers with citric acid as the top diagnostic compound (VIP: 22.09), followed by isonuatigenin 3-[rhamnosyl-(1→2)-glucoside] (VIP: 13.74). PS exhibited 38 exclusive markers led by dihydroprudomenin (VIP: 14.54), a characteristic flavonoid. PC displayed 39 exclusive markers including trehalose (VIP: 13.84) and D-(+)-raffinose (VIP: 11.40). PO contained 20 exclusive markers with 7,8-dihydromethanopterin as the primary diagnostic compound (VIP: 12.57). These species-specific markers enable systematic differentiation through a multi-tier classification approach, with high VIP exclusive markers (Tier 1) providing definitive identification, discriminative markers (Tier 2) offering supporting evidence, and chemical class signatures (Tier 3) serving as confirmatory validation (see Supplementary Protocol S1 and Table S3).

## 4. Discussion

Recently, with the increased awareness of health improvement, PR has become a significant research hotspot. In the *Chinese Pharmacopoeia*, dry rhizomes of the *Liliaceae Polygonatum* genera primarily include PK, PS, PC, and PO [5]. These four PRs have been considered a “medicine food homology” by the National Health Commission of the People’s Republic of China [4]. PR contains various functional polysaccharides, steroids, alkaloids, nucleosides, and peptides [6]. Studies of the clinical and molecular mechanisms of Chinese medicines have indicated that PR has good antihyperglycemic [7,8], anti-inflammatory [9], immune regulatory, blood lipid-lowering, and antitumor effects, with potential pharmaceutical and medical value [10]. More importantly, as far as we know, there is a lack of research comparing the metabolite profiles of PK, PS, PC, and PO, leading to obstacles demonstrating the differences in their nutritional and medical values. Therefore, in the processes of clinical disease treatment, new drug research, and functional food development, the four metabolites of PK, PS, PC, and PO were unclear, and the selection of raw materials was thus arbitrary. Here, we used an untargeted metabolomics analysis using a liquid-mass spectrometry system comprising a UHPLC ultra-performance liquid phase tandem QE high-resolution mass spectrometer. We initially determined the differential metabolites between the four PRs using statistical analysis and further explored the relationships among the metabolites through correlation and clustering analyses.

Overall, 2360, 2336, 2381, and 2355 unique metabolites were detected in PK, PS, PC, and PO, respectively. The metabolites of the four PRs significantly overlapped, PK, PS, PC, and PO contained 36, 26, 10, and 5 unique components, respectively. PC had more unique characteristics than the other three varieties. The PCA results showed that metabolites varied among PK, PS, PC, and PO as they were distributed in different areas (Figure 3). We observed good separation among the four samples, indicating significantly differential metabolites (Figure 4). The specific metabolites of these four species could help distinguish different varieties and provide a basis for clinical disease treatment, new drug research, and functional food development.

Our systematic marker analysis provides practical tools for species authentication and quality control. The 168 species-specific metabolites identified offer robust chemical fingerprints for differentiation, with high VIP exclusive markers such as citric acid for PK, dihydroprudomenin for PS, trehalose for PC, and 7,8-dihydromethanopterin for PO enabling reliable species verification. We developed a multi-tier classification system integrating exclusive markers (Tier 1), discriminative markers (Tier 2), and chemical class signatures (Tier 3) with a quantitative scoring algorithm that provides objective confidence assessments. This systematic approach addresses adulteration concerns and can be implemented in quality control laboratories using standard LC-MS/MS platforms (detailed protocol in Supplementary Material).

A total of 61, 56, 61, and 57 carbohydrates were identified in PK, PS, PC, and PO, respectively. Polysaccharides are the most crucial component in the *Liliaceae Polygonatum mill*, and a significant index for evaluating PR quality. The polysaccharide content ranges between 4.47% and 21.34% [17]. Among 33 production areas, the polysaccharide contents were the highest (14.09%) and lowest (2.23%) in *Polygonatum* from Shaoguan City, Guangdong, and in Xinfeng County, Jiangxi Province, respectively [25]. Li et al. (2018) found that the contents of PS solid polysaccharides solubilized in hot buffer, chelating agent, diluted alkali, and concentrated alkali were 7.02%, 17.73%, 28.34%, and 8.68%, respectively [26]. All of them contained rhamnose, arabinose, xylose, mannose, glucose, galactose, and galacturonic acid. PR polysaccharides significantly reduce blood glucose and serum glycosylated hemoglobin concentrations in mouse models of diabetes [27], whereas they increased streptozotocin-induced insulin expression in diabetic rats [28]. Low doses of *Polygonatum* polysaccharides exerted significant inhibitory effects on H22 solid tumors, whereas medium and high doses significantly prolonged the survival of S180 mice bearing ascites tumors [29,30]. In another study, *Polygonatum* polysaccharides upregulated the expression of basic fibroblast growth factors and bone morphogenetic proteins [31]. Considering these findings, our results revealed that the polysaccharide components varied between different PR species. The differences in metabolomic profile also showed distinctive nutritional and medicinal values among these PR strains. Our results provide a theoretical reference for the qualitative polysaccharide regulation.

A total of 33, 32, 29, and 30 steroids were identified in the PK, PS, PC, and PO, respectively. We identified different steroids among the *Polygonatum* species. Steroids are crucial for health maintenance and for treating certain diseases. For example, estrogen promotes follicle development and the formation and maintenance of the corpus luteum [32,33]. During pregnancy, estrogen stimulates breast growth by regulating the expression of the oxytocin receptor gene and promotes uterine blood flow to maintain a normal pregnancy [34,35]. Estrogen also functions in regulating bone resorption [36], cardiovascular disease prevention [37], neuroprotection against Alzheimer’s and Parkinson’s diseases, death due to stroke [38], multiple cytokine secretion [39], protection against autoimmune diseases [40], appetite and eating behavior [41], and lipid and carbohydrate metabolism [42]. Our results revealed that the estrone was only expressed in PK. We speculated that PK may be effective in treating diseases caused by estrogen deficiency. Estrogen can also serve as a marker for identifying PK. Taken together, our findings may shed light on the future application of various PRs in the treatment of different diseases. A total of 10, 12, 12, and 11 alkaloids were identified in PK, PS, PC, and PO, respectively. Alkaloids exert therapeutic effects on cardiovascular and cerebrovascular diseases, hyperlipidemia, hypertension, hepatitis, and myasthenia gravis [43]. For example, xanthoplanine weakens the polarization and inflammatory response of macrophages to the M1 phenotype by disrupting the CrkL-STAT5 complex [44]. A dextromethorphan trial revealed at least a 20% reduction in baseline pain in six participants [45]. We detected xanthoplanine and dextromethorphan only in PS. The present study revealed the metabolism of four rhizome alkaloids, thus providing a reference for the treatment of inflammatory responses and baseline pain.

Furthermore, we performed a systematic KEGG pathway analysis to reveal differential pathways in PK, PS, PC, and PO. Interestingly, results showed that differential enriched pathways between four PRs. We mapped the PK, PS, PC, and PO metabolites to the KEGG database to determine the metabolic pathways in the four *Polygonatum* species (Figure 8). We identified several amino acid metabolic pathways, including glycine, serine, and threonine metabolism, as well as aminoacyl tRNA, valine, leucine, and isoleucine biosynthetic pathways. It functions as a neurotransmitter in the central nervous system and also has broad-spectrum anti-inflammatory, cytoprotective, and immunomodulatory effects [46,47,48]. Serine is the precursor of cysteine and glycine and plays a role in many crucial biological pathways, such as glycolysis, purine synthesis, carbon metabolism, the methionine cycle, and glutathione synthesis [49]. Leucine, valine, threonine, and isoleucine are essential amino acids that cannot be independently synthesized by humans and are thus acquired from food. The expression of amino acid metabolites was high in PK and PS but low in PO. These results indicated that the four *Polygonatum* species exert different physiological activities because of the variation in their nutrient composition. Therefore, the key metabolites observed in four *Polygonatum* species may be attributed to different genetic backgrounds. Taking all together, our findings might shield light on the future application of various PRs in the treatment of different diseases, new drug research, and functional food development.

Estrone, which was uniquely expressed in PK in our study, has important hormonal activities. Estrone (E1) is one of three major endogenous estrogens and binds to estrogen receptors (Erα and Erβ), though it exhibits weaker estrogenic activity compared to estradiol [https://www.ncbi.nlm.nih.gov/books/NBK549797/, accessed on 14 October 2025] [https://www.ncbi.nlm.nih.gov/books/NBK538260/, accessed on 14 October 2025]. In postmenopausal women, estrone becomes a principal circulating estrogen and has been positively correlated with bone mineral density [https://pmc.ncbi.nlm.nih.gov/articles/PMC8836058/, accessed on 14 October 2025]. Estrogen deficiency following menopause is associated with accelerated bone loss and increased risk of osteoporosis, and hormone therapy effectively prevents postmenopausal bone loss [https://www.ncbi.nlm.nih.gov/pmc/articles/PMC8836058/, accessed on 15 October 2025] [https://www.ncbi.nlm.nih.gov/pmc/articles/PMC2957171/, accessed on 15 October 2025]. These findings suggest that PK may have potential therapeutic applications in estrogen-related conditions, particularly for menopausal women experiencing estrogen deficiency, bone loss, and related symptoms. The unique presence of estrone in PK also provides a distinctive biomarker for quality control and species authentication.

The alkaloids xanthoplanine and dextromethorphan, which were detected only in PS in our study, possess notable anti-inflammatory and analgesic properties with important therapeutic implications. Xanthoplanine significantly reduces M1 macrophage polarization and promotes M2 polarization, with marked decreases in inflammatory cytokines when macrophages are pretreated with xanthoplanine compared to those induced by lipopolysaccharide and interferon-γ [https://pubmed.ncbi.nlm.nih.gov/32142888/, accessed on 14 October 2025]. The mechanism involves disrupting the CrkL-STAT5 complex, thereby attenuating the M1 phenotypic switch and macrophage inflammation [https://pubmed.ncbi.nlm.nih.gov/32142888/, accessed on 15 October 2025]. Regarding dextromethorphan, meta-analyses of randomized controlled trials demonstrate that perioperative dextromethorphan use significantly reduces postoperative opioid consumption at 24–48 h and decreases pain scores at multiple time points [https://www.ncbi.nlm.nih.gov/pmc/articles/PMC4755866/, accessed on 13 October 2025] [https://pubmed.ncbi.nlm.nih.gov/26587683/, accessed on 14 October 2025]. These findings suggest that PS may be particularly valuable for treating inflammatory conditions and managing pain, distinguishing it from the other three PR species in terms of potential clinical applications.

## 5. Conclusions

Our study identified a wide range of metabolic profiles of PK, PS, PC, and PO, respectively, through untargeted, a liquid–mass spectrometry system comprising a UHPLC ultra-performance liquid phase tandem QE high-resolution mass spectrometer based on metabolomics. To our knowledge, this is the first attempt to study PK, PS, PC, and PO metabolites and metabolic pathways, in addition to analyses of differentially expressed metabolites between PK, PS, PC, and PO. Our results provide a comprehensive description of the overall metabolic profiles of PK, PS, PC, and PO, facilitating research on the functions and pharmacodynamics of PR species at the metabolomic level. Thus, our findings provide both a theoretical basis and practical diagnostic tools for qualitative evaluation, species authentication, and comprehensive utilization of PR.

## Figures and Tables

**Table 1:** Statistical parameters for PCA, PLS-DA, and supervised OPLS-DA analyses.

Group	Type	R^2^X (cum)	R^2^Y (cum)	Q2 (cum)	R2	Q2
PS/PO	PCA	0.509				
PS/PO	PLS	0.732	0.996	0.969		
PS/PO	OPLS	0.732	0.996	0.968	0.824	−0.375
PC/PO	PCA	0.562				
PC/PO	PLS	0.681	0.996	0.87		
PC/PO	OPLS	0.681	0.996	0.859	0.903	−0.389
PK/PO	PCA	0.557				
PK/PO	PLS	0.619	0.996	0.946		
PK/PO	OPLS	0.619	0.996	0.923	0.898	−0.39
PS/PK	PCA	0.594				
PS/PK	PLS	0.699	0.997	0.924		
PS/PK	OPLS	0.699	0.997	0.912	0.906	−0.296
PC/PK	PCA	0.561				
PC/PK	PLS	0.636	0.998	0.938		
PC/PK	OPLS	0.636	0.998	0.909	0.908	−0.329
PS/PC	PCA	0.58				
PS/PC	PLS	0.7	0.995	0.883		
PS/PC	OPLS	0.7	0.995	0.884	0.854	−0.324

## Data Availability

All data generated or analyzed during this study are included in this published article and its supplementary information files.

## Supplementary Materials

The following supporting information can be downloaded at: https://www.mdpi.com/article/10.3390/metabo15110682/s1, Supplemental Table S1: Unique metabolite features were detected among all four different *Polygonati rhizome*; Supplemental Table S2: Metabolites differentially expressed in four different *Polygonati rhizome*; Supplemental Table S3: Chemical class of top diagnostic metabolites in four different *Polygonati rhizome*; Supplementary Protocol S1: Step-by-step authentication protocol for *Polygonati* rhizoma.

## Abbreviations

The following abbreviations are used in this manuscript:

KEGG	Kyoto Encyclopedia of Genes and Genomes
MS	Mass spectrometry
OPLS-DA	Orthogonal partial least squares discriminant analysis
PC	*Polygonatum cyrtonema*
PCA	Principal component analysis
PK	*Polygonatum kingianum*
PLS-DA	Partial least squares discriminant analysis
PO	*Polygonatum odoratum*
PR	*Polygonati* rhizoma
PS	*Polygonatum sibiricum*
