# Supplementary material for: Metabolic Signatures of Four Polygonatum Rhizoma Species Mapped Using Untargeted Metabolomics
Source: Metabolites. 2025 Oct 22;15(11):682. doi: 10.3390/metabo15110682 (PMC12654418; doi:10.3390/metabo15110682)
Supplement: Supplementary file 1 [file metabolites-15-00682-s001.zip › Supplementary Protocol S1.pdf]

Supplementary Protocol S1:

STEP-BY-STEP AUTHENTICATION PROTOCOL FOR POLYGONATUM RHIZOMA:

STEP 1: SAMPLE PREPARATION AND METABOLOMICS ANALYSIS

- Extract metabolites from Polygonatum rhizoma sample
- Perform untargeted LC-MS metabolomics analysis
- Generate metabolite profile with VIP scores

STEP 2: TIER 1 SCREENING - EXCLUSIVE MARKERS (Definitive Identification)

Check for presence of exclusive markers (VIP > 2):

PK (P. kingianum) - Look for:

- Citric acid (VIP: 22.09)
- Isonuatigenin 3-[rhamnosyl-(1->2)-glucoside] (VIP: 13.74)
- PA(18:4/20:4) derivatives (VIP: 5.43)

PS (P. sibiricum) - Look for:

- Dihydroprudomenin (VIP: 14.54)
- 5,7,8-trihydroxy-3-methoxy-2-(3-methoxyphenyl) derivative (VIP: 12.97)
- Zearalenone 4-sulfate (VIP: 7.27)

PC (P. cyrtonema) - Look for:

- 3,4-Dihydro-2H-1-benzopyran-2-one (VIP: 10.25)
- Uvaricin (VIP: 4.12)
- L-Aspartic Acid (VIP: 3.13)

PO (P. odoratum) - Look for:

- Idebenone Metabolite (VIP: 2.64)
- 8-Butanoylneosolaniol (VIP: 2.64)

→ If exclusive markers detected: HIGH CONFIDENCE identification

STEP 3: TIER 2 CONFIRMATION - DISCRIMINATIVE MARKERS (Supporting Evidence)

If Tier 1 is ambiguous, check for high VIP discriminative markers:

- Count number of species-associated markers with VIP > 3
- Species with most matched markers is likely identity

→ Confidence: MEDIUM-HIGH

STEP 4: TIER 3 VALIDATION - CHEMICAL CLASS SIGNATURES (Additional Support)

Compare chemical class distribution:

PK: High lipids (27.5%), moderate unclassified (25.8%)

PS: High unclassified (34.3%), moderate lipids (18.5%), organic acids (13.9%)
PC: High unclassified (31.0%), moderate lipids (17.7%)
PO: High unclassified (34.0%), moderate lipids (20.8%)

→ Use as confirmatory evidence

STEP 5: CALCULATE CONFIDENCE SCORE

- Apply scoring system based on detected markers
- Compare top 2 species scores
- Score ratio > 2.0: HIGH confidence
- Score ratio 1.5-2.0: MEDIUM confidence
- Score ratio < 1.5: LOW confidence (need additional analysis)

QUALITY CONTROL RECOMMENDATIONS:

1. Always run biological replicates ($n \geqslant 3$)
2. Include authenticated reference samples
3. Use consistent extraction and analysis protocols
4. Validate with orthogonal methods if confidence is LOW
5. Consider regional/seasonal variations in metabolite profiles
